# Molecular and cellular processes connecting type 2 diabetes to Alzheimer’s disease, focusing on oxidative stress, metabolic dysfunction, and neurodegeneration

**DOI:** 10.1590/1678-4685-GMB-2025-0233

**Published:** 2026-07-10

**Authors:** Renata M.S. Ono, Jessica E.B.F. Lima, John A.S.P. Lira, Fernanda C.A. Sanches, Larissa O. Piassi, Luisa B.V. Coelho, Natalia C.S. Moreira, Elza T. Sakamoto-Hojo

**Affiliations:** 1 Universidade de São Paulo, Faculdade de Medicina de Ribeirão Preto, Departamento de Genética, Ribeirão Preto, SP, Brazil.; 2 Universidade de São Paulo, Faculdade de Filosofia, Ciências e Letras de Ribeirão Preto, Departamento de Biologia, Ribeirão Preto, SP, Brazil.; 3 University of California San Diego, Sanford Stem Cell Institute, Integrated Space Stem Cell Orbital Research Center, La Jolla, CA, United States .

**Keywords:** Type 2 diabetes, Alzheimer’s disease, insulin resistance, oxidative stress, neurodegeneration

## Abstract

Advancing age has been associated with an imbalance between a gradual increase in reactive oxygen species (ROS) production and a decline in the efficiency of antioxidant defense mechanisms. Due to the fact that organisms are constantly exposed to endogenous and exogenous ROS sources, this condition of oxidative stress promotes a systemic pro-oxidant state that, in conjunction with many other risk factors, progressively worsens throughout life, triggering the development of chronic diseases, such as type 2 diabetes (T2D) and Alzheimer’s disease (AD). These diseases have emerged as closely interconnected conditions that share some common molecular and cellular mechanisms. In this review, on the light of literature data, we explore the interconnection between several pathological processes implicated in both diseases, focusing on a series of cellular and molecular alterations, from metabolic dysfunction to neurodegeneration (including insulin resistance, oxidative stress, mitochondrial and endoplasmic reticulum dysfunction, among others). We highlight how the connection between those processes may culminate in the development of neurodegeneration and disease progression. Furthermore, we discuss the challenges and limitations to model those diseases, emphasizing some aspects of the current knowledge in terms of providing new perspectives to the development of multi-target therapeutic approaches.

## Introduction

Metabolic diseases have been strongly associated with dementia and neurodegeneration. In fact, accumulating evidence shows that individuals with metabolic dysregulation, as mainly observed in obesity and diabetes mellitus, are more susceptible to develop dementia and neurodegenerative diseases. Epidemiological studies conducted in a nationwide of individuals (above the age of 65 years) in Denmark showed an association between type 2 diabetes (T2D) and major subtypes of dementia, including vascular dementia and Alzheimer’s Disease (AD) (Thomassen et al., 2020). 

T2D is the most prevalent type of diabetes, accounting for approximately 90% of all 589 million cases recorded worldwide (International Diabetes Federation, 2025). It is estimated that by 2050, the number of cases will increase to 852 million. Even though T2D has been most diagnosed in patients over 50 years of age, the number of cases in younger individuals has increased in recent years, mainly due to a more sedentary lifestyle and obesity (International Diabetes Federation, 2025). According to a longitudinal cohort study, diabetes onset at a younger age was significantly associated with a higher risk of subsequent dementia. In this study, the authors performed a median follow-up of 31.7 years in 1,710 cases of diabetes and 639 cases of dementia (Barbiellini et al., 2021).

T2D is characterized by hyperglycemia resulting from insulin resistance (IR), which is a condition that plays an early role in its pathogenesis and progression to higher glycemic levels (Lowell and Schulman, 2005). The persistence of insulin resistance for years culminates in pancreatic β cell dysfunction and insufficient insulin production (James et al., 2021; Lee et al., 2021). Although early stages of T2D are often asymptomatic, ensuing in delayed diagnosis and treatment, as long as hyperglycemia becomes chronic throughout life, patients develop several complications (such as retinopathy, nephropathy, neuropathy, diabetic foot), and other associated diseases, which can be accompanied by a 2- to 5-fold increased risk of developing AD (Gudala et al., 2013; International Diabetes Federation, 2025).

AD is the most common form of dementia, accounting for approximately 60-80% of all cases, and currently affecting over 55 million people worldwide (Alzheimer’s Disease International, 2022). The main neuropathological markers of the disease include the formation of intracellular neurofibrillary tangles (NFTs) of hyperphosphorylated tau protein and the aggregates of the amyloid-beta (Aβ) peptide in extracellular oligomers, both of which contribute to chronic neuronal damage and dysfunction (Alzheimer’s Disease International, 2022; Lima et al., 2022, review article).

The adult brain is a highly energy-demanding organ, consuming approximately 25% of total body glucose under both awake and resting states (Chen and Zong, 2013). Advances in neuroimaging technology have allowed researchers to investigate the relationship between brain energy metabolism and AD progression *in vivo,* through positron emission tomography (PET). This method uses the tracer F-fluorodeoxyglucose (FDG), which is a marker of glucose transport and glycolysis phosphorylation in the brain (de Leon et al., 1983). Using this method, Mosconi (2005) demonstrated a correlation between progressive reductions in cerebral glucose metabolism and the severity of AD symptoms. Notably, impairments in cerebral glucose utilization caused by T2D have been shown to precede cognitive decline and neuropathological changes decades before the first symptoms of AD (Talbot et al., 2012; An et al., 2017; Hoyos et al., 2022).

Remarkably, insulin signaling regulates key brain processes related to memory formation and synaptic plasticity, in part through downstream regulation of glycogen synthase kinase 3 beta (GSK3-β) (Jolivalt et al., 2008). Post-mortem analyses further reveal that brains from patients with T2D exhibit an AD-like pattern of reduced cerebral glucose metabolic rate in frontal, parietotemporal, and cingulate regions (Baker et al., 2011). Impaired insulin pathway increases oxidative stress, Aβ accumulation and tau protein phosphorylation in patients with T2D (Sims-Robinson et al., 2010; Hoyos et al., 2022), reinforcing that impaired glucose metabolism in the brain is such an important factor that has been termed as “type 3 diabetes” (or cerebral insulin resistance) (Lester-Coll et al., 2006).

Transcriptomic studies showed altered expression profiles of diabetes mellitus (DM)-related genes in AD brains, which were independent of peripheral DM-related abnormalities. Their results indicated that altered expression of genes related to DM in AD brains is a result of AD pathology, which may thereby be exacerbated by peripheral insulin resistance or DM (Hokama et al., 2014). Another report on transcriptome analyses identified shared molecular signatures between AD and T2D datasets, through integrated analysis of temporal cortex gene expression data; the authors reported 16 common differentially expressed genes (DEGs) between the two diseases, and the biological processes were found related to apoptosis, autophagy, inflammation, and hemostasis (Shu et al., 2022). Important contributions have been provided by high-throughput proteomic profiling in an attempt to clarify the molecular and mechanistic links between AD and T2D (reviewed by Chen et al., 2025). According to the authors, although proteomic studies emphasize strong links between AD and T2D, a deeper investigation is still required to identify and clarify molecular mechanisms and potential therapeutic targets.

Thus, substantial evidence generated through numerous research tools and approaches supports a link between T2D and AD, but mechanistic approaches are still not sufficient to point to causal associations and elucidate all relevant processes implicated in both pathologies. In spite of this, there is plenty of literature information indicating the association and connection between those two diseases (Table 1 and 2A). Collectively, all findings added relevant information to strengthen molecular and cellular shared pathways linking T2D to AD.

**Table 1 -:** Research studies that correlate Metabolic dysfunction (diabetes-related) and AD.

Author (Year) Journal	Title	Model / Population	Diabetes / Metabolic Factor	AD / Cognitive Findings
Shu et al. (2022) *Gene*	Detection of molecular signatures and pathways shared by Alzheimer’s disease and type 2 diabetes	Humans (Bioinformatics / Dataset analysis)	Shared molecular pathways between T2D and AD.	Identified common genes and molecular shared mechanisms between T2D and AD.
Thomassen et al. (2020) *Epidemiol Psychiatr Sci*	Type-2 diabetes and risk of dementia: observational and Mendelian randomisation studies in 1 million individuals	Humans (Observational and Mendelian Randomization)	T2D confirmed	Observational data showed T2D increased dementia risk; Mendelian randomization did not support a causal effect
Thubron et al. (2019) *Sci Rep*	Regional mitochondrial DNA and cell-type changes in post-mortem brains of non-diabetic Alzheimer’s disease are not present in diabetic Alzheimer’s disease	Humans (Post-mortem: Diabetic AD vs Non-Diabetic AD)	Comparison of Diabetic vs Non-Diabetic	Neuropathology differs between Diabetic-AD and Non-Diabetic AD
Santiago et al. (2019) *Front Neurosci*	Transcriptomic and network analysis highlight the association of diabetes at different stages of Alzheimer’s disease	Humans (Blood: T2D vs. MCI and AD - Transcriptomic Analysis)	Diabetes-associated gene expression patterns identified	Shared pathways and molecular networks between T2D vs. MCI and T2D vs. AD, specially PI31K/AKT
An et al. (2017) *Alzheimers Dement*	Evidence for brain glucose dysregulation in Alzheimer’s disease	Humans (AD patients)	Brain glucose dysregulation	Glucose dysregulation correlates with severity of AD pathology.
Barbiellini Amidei et al. (2021) *JAMA*	Association between age at diabetes onset and subsequent risk of dementia	Humans (Longitudinal Cohort)	T2D confirmed	Younger age at T2D onset is associated with a higher risk of dementia.
Hokama et al. (2014) *Cereb Cortex*	Altered expression of diabetes-related genes in Alzheimer’s disease brains: the Hisayama study	Humans/AD mouse (Post-mortem brains)	Alterations in diabetes-related genes in AD brains.	Altered metabolic gene expression is a distinct feature of AD pathology in the hippocampus.
Gudala et al. (2013) *J Diabetes Investig*	Diabetes mellitus and risk of dementia: A meta‐analysis of prospective observational studies	Humans (Meta-analysis)	T2D confirmed	T2D patients have a significantly higher risk of developing dementia/AD.
Talbot et al. (2012) *J Clin Invest*	Demonstrated brain insulin resistance in Alzheimer’s disease patients is associated with IGF-1 resistance, IRS-1 dysregulation, and cognitive decline	Humans/AD Mouse (Post-mortem brains)	Brain IR; IGF-1 and IRS-1 dysregulation.	Brain IR appears to be an early feature of AD, potentially triggered by Aβ oligomers promoting cognitive decline.
Baker et al. (2011) *Arch Neurol*	Insulin Resistance and Alzheimer-like Reductions in Regional Cerebral Glucose Metabolism for Cognitively Normal Adults With Prediabetes or Early Type 2 Diabetes	Humans (Prediabetes or early T2D)	IR associated with reduced CMRglu	AD-like hypometabolism patterns in cognitively normal IR adults
Ott et al. (1999) *Neurology*	Diabetes mellitus and the risk of dementia: The Rotterdam study	Humans (Epidemiological Study)	T2D confirmed	Strong association between T2D and AD; DM almost doubled the dementia risk
Jolivalt et al. (2008) *J Neurosci Res*	Defective insulin signaling pathway and increased GSK-3 activity in the brain of diabetic mice: parallels with Alzheimer’s disease and correction by insulin	Mice (Diabetic model)	Defective brain insulin signaling	DM mice shows similar alterations to AD models brains; Insulin treatment improved the alterations.

The studies are listed based on model/population and year, starting from the most recent studies in humans, followed by in vivo and in vitro studies. AD: Alzheimer’s Disease; T2D: Type 2 Diabetes; DM: Diabetes Mellitus; MCI: Mild Cognitive impairment; IR: Insulin resistance; CMRglu: Cerebral Metabolic Rate of Glucose.

In the following topics, we focus on several mechanisms underlying important molecular and physiological alterations, reinforcing an interplay between T2D and AD to illustrate sequential processes, from metabolic dysfunction to neurodegeneration, focusing on oxidative stress as an intersection point among those alterations. We also discuss the challenges, advancements, and limitations in modeling those diseases.

## ROS generation, oxidative stress and defense mechanisms

Reactive Oxygen Species (ROS) encompass all unstable metabolites derived from oxygen (O₂), including highly reactive free radicals and non-radical molecules (Averill-Bates, 2024). Interestingly, ROS play a dual role in the organism; under normal conditions, low/moderate ROS levels act as important signaling molecules, which participate in several cellular processes, including gene expression and cell differentiation; in contrast, when ROS production overwhelms the cellular antioxidant capacity, the consequence is a significant damage to cellular components, ultimately leading to the state of oxidative stress (Zhang et al., 2022 a ; Ekundayo et al., 2024).

In living cells, there is a constant endogenous ROS formation as byproducts of their metabolism, primarily due to the leakage of electrons from the mitochondrial respiratory chain (Zhang et al., 2022 a ). These electrons can react with oxygen, forming superoxide anions (O₂^•-^), which is the most abundant form of ROS. Other forms of ROS include the hydrogen peroxide (H₂O₂), the hydroxyl radical (•OH), and the singlet oxygen (^1^O_2_) (Zorov et al., 2014; Tönnies and Trushina, 2017). In addition, through the interaction with nitric oxide synthase, O₂^•-^ can also form reactive nitrogen species (RNS), such as nitric oxide (NO), which can be converted into the highly reactive peroxynitrite (ONOO^-^) (Valko et al. 2007). ROS can also be generated from exogenous sources, such as chemical and physical agents, environmental contaminants, and products generated through lifestyle. Together, the excessive amount of ROS can cause oxidative damage to biomolecules, such as lipids, proteins, carbohydrates, and nucleic acids (Averill-Bates, 2024). In this scenario, oxidative stress may trigger a series of harmful consequences to the whole organism.

In general, organisms must cope with an imbalance between the production of ROS and the efficiency of antioxidant defense mechanisms (Ramsey et al., 2007). DNA is one of the primary targets of ROS, and oxidative DNA damage causes multiple types of DNA lesions that can lead to mutagenesis and cell death (Poetsch, 2020). The accumulation of these lesions has been implicated in the progression of both metabolic and neurodegenerative disorders. For instance, even a slight elevation in blood glucose levels in prediabetic patients increases oxidative DNA damage, as evidenced by elevated serum 8-OHdG concentrations (Al-Aubaidy and Jelinek 2011). Remarkably, a seven-day hospitalization period aimed at improving glycemic control in T2D patients was sufficient to significantly reduce DNA damage levels (Xavier et al., 2014). Additionally, hyperglycemic T2D patients showed several differentially expressed genes related to inflammation, DNA repair, ROS production and antioxidant defense, reinforcing the association between hyperglycemia and increased DNA damage (Xavier *et al*., 2015). 

Moreover, alterations in DNA repair mechanisms and cell cycle regulation have been reported in patients with AD (Weissman et al., 2007). In AD, marked DNA repair deficiencies have been identified, including limited base damage processing by DNA glycosylases and reduced DNA synthesis by DNA polymerase β; similar defects were also observed in amnestic mild cognitive impairment (MCI) brains, where they correlated with the amount of NFTs (Weissman *et al*., 2007). Consistently, other studies have also found that patients with T2D and patients with AD exhibit elevated oxidative DNA damage and reduced DNA repair capacity (Blasiak et al., 2004; Xavier et al., 2014). These alterations, together with a sustained oxidative stress condition, lead to a chronic accumulation of DNA damage, both nuclear and mitochondrial (mtDNA), progressively compromising cellular function and promoting the development of metabolic and degenerative diseases, particularly T2D and AD, respectively (Ahmad et al., 2017; Shu et al., 2020; Lima et al., 2022). 

The antioxidant system counteracts the damaging effects of ROS, constituting a natural defense mechanism mainly composed of non-enzymatic components and antioxidant enzymes, such as superoxide dismutases (SODs), that catalyze the conversion of O₂^•-^ into H₂O₂; glutathione peroxidases and catalases, that subsequently reduce H₂O₂ molecules into H₂O and O₂. However, H₂O₂ can also react with metal ions, especially Fe²⁺, through the Fenton reaction, forming •OH, which is the most reactive form of ROS, and cannot be enzymatically neutralized by the antioxidant system (Brieger et al., 2012; Poprac et al., 2017). 

The antioxidant response involve the participation of a key transcriptional regulator, the Nuclear Factor Erythroid 2-Related Factor 2 (NRF2), which activates several antioxidant genes in response to oxidative stress (Fão et al., 2019). The primary negative regulator of NRF2 is the Kelch-Like ECH-Associated Protein 1 (KEAP1). In response to oxidative stress, NRF2 dissociates from KEAP1 in the cytosol and translocates to the nucleus, where it promotes the transcription of antioxidant genes (Suzuki et al., 2023). Another regulatory process involves the protein GSK3-β, which phosphorylates NRF2, leading to its ubiquitination and subsequent degradation (Rada et al., 2011). GSK3-β, in turn, is inhibited by the protein kinase AKT. Thus, the AKT pathway positively regulates NRF2 activation (Cuadrado et al., 2018). Interestingly, NRF2 activation was found reduced in AD (Cazzaro et al., 2023), and in fact, other authors reported that the antioxidant response is compromised in AD, favoring the pro-oxidant state and the subsequent generation of cell damage (Ramsey et al., 2007; Rojo et al., 2017).

Therefore, there is extensive evidence supporting the hypothesis that chronic oxidative stress condition in T2D can cause neuronal DNA damage that might be inefficiently repaired, thereby promoting cell death and neurodegeneration, leading to an increased risk of developing AD. Given that oxidative stress represents a significant source of cellular and molecular damage throughout life, special attention should also be directed toward the molecular mechanisms of cellular defense, focusing on antioxidant systems, DNA damage repair, and other processes underlying sequential pathophysiological changes and disease outcome.

## Oxidative stress and mitochondrial dysfunction

Mitochondrial DNA (mtDNA) is highly susceptible to the effects of oxidative stress, due to the lack of protective histones, the location close to the primary cellular source of ROS in the inner membrane and as a consequence of inefficient DNA repair mechanisms compared to nuclear DNA. High intracellular ROS levels can cause irreversible damage to mtDNA, which may result in mutations in essential genes for mitochondrial function (Anderson et al., 2020; Steffan et al., 2025). These alterations can lead to cellular bioenergetic deficits, disrupted synthesis of essential molecules, and abnormal mitochondrial signaling, as well as further ROS production, establishing a vicious cycle in which elevated ROS exacerbate mitochondrial dysfunction, further promoting additional ROS production (Shu et al., 2020). 

Under conditions of metabolic overload, such as high glucose levels, ROS production is further amplified, aggravating mitochondrial dysfunction, inflammation and exacerbating cellular damage (reviewed in Lima et al., 2022; Wang et al., 2026). One indicator of mitochondrial function is the number of mitochondria within cells, which can vary according to physiological conditions. Altered mitochondrial mass has been associated with changes in mtDNA content, and indeed, variations in mtDNA levels have been reported in both peripheral tissues of T2D patients and in the brains of AD patients (Malik et al., 2015; Thubron et al., 2019). In the brains of pre-diabetic and T2D patients, IR and hyperglycemia have been associated with cognitive decline and neuronal death via autophagy, apoptosis, and ferroptosis, and are considered a common link between T2D and AD (Baker et al., 2011; Bhatia and Sharma, 2021; Tang et al., 2022). 

Under conditions of high blood glucose levels in T2D, the consequence is the formation of advanced glycation end-products (AGEs), which result from non-enzymatic reactions that cause the irreversible glycation of amino residues in proteins and lipids (Jiang et al., 2022). The interaction of AGEs with their receptor (RAGE) triggers oxidative stress and inflammation. Concurrently, oxidative stress upregulates the expression of RAGE, creating a positive feedback loop (Byun et al., 2017; Wu et al., 2021). This AGE-RAGE signaling leads to mitochondrial dysfunction and inflammation via activation of the nuclear factor kappa B (NF-κB) pathway, leading to Aβ deposition and tau hyperphosphorylation (Gasparotto et al., 2018; Jiang *et al*., 2022). The pro-oxidant state observed in T2D patients promotes protein modifications, including the pathological hallmarks of AD, that are Aβ plaques and NFTs (Sultana et al., 2011). The Aβ peptide has been reported to disrupt the mitochondrial electron transport chain, resulting in electron leakage and excessive ROS production. This dysfunction also compromises mitochondrial dynamics and causes damage to mitochondrial membranes, ultimately promoting organelle fragmentation (Chen and Zhong, 2014). In addition, there is accumulated evidence supporting the hypothesis that IR in humans arises from defects in mitochondrial fatty acid oxidation, which in turn lead to increased intracellular fatty acid metabolites that disrupt insulin signaling (Lowell and Schulman, 2005). 

In fact, mitochondrial dysfunction is a crucial factor in AD and age-related diseases (Bhatia and Sharma, 2021), but the underlying biological processes and comparisons between normal aging and age-related neurodegenerative diseases still remain to be clarified. In spite of this, oxidative stress and mitochondrial dysfunction, which are connected to impaired insulin signaling, have been considered fundamental pathological processes in both T2D and AD (Table 2), thus reinforcing the link between the two diseases.

**Table 2 -:** Research studies that correlate: (A) Oxidative stress, metabolic dysfunction (diabetes-related) and AD/neurodegeneration; (B) Oxidative stress and AD/neurodegeneration; (C) Oxidative stress and metabolic dysfunction (diabetes-related).

Author (Year) Journal	Title	Model / Population	Diabetes / Metabolic Factor	Oxidative Stress Findings	AD / Cognitive Findings
A) Oxidative stress, metabolic alterations (diabetes-related) and AD/neurodegeneration					
Hoyos et al. (2022) *Diabetes Res Clin Pract*	Brain oxidative stress and cognitive function in older adults with diabetes and pre-diabetes who are at risk for dementia	Humans (DM and pre-DM)	Pre-DM and T2D status confirmed	­GSH associated with ­ glucose/HbA1c	Impaired memory and executive dysfunction in diabetic/pre-diabetic groups at risk of dementia
Hatanaka et al. (2016) *Geriatri Gerontol Int*	Peripheral oxidative stress markers in diabetes-related dementia	Humans (Blood and urine: DrD, AD+DM and AD)	DM status confirmed	­ Peripheral oxidative stress markers ¯antioxidant	Correlation between oxidative stress markers and dementia
Lee et al. (2013) *J Alzheimers dis Parkinsonism*	CSF and Brain Indices of Insulin Resistance, Oxidative Stress and Neuro-Inflammation in Early versus Late Alzheimer’s Disease	Humans (AD CSF, VF and Brain tissue)	CSF/Brain indices of IR	Oxidative stress (­8-OHdG)	Linked metabolic/oxidative/inflammatory axes with AD severity
Wang et al. (2026) *Free Rad Biol Med*	Genistein ameliorates glucose-induced β-amyloid toxicity, oxidative stress, and aging in the C. elegans model of Alzheimer’s disease	*C. elegans* (AD model)	Glucose-induced toxicity	Glucose ­ oxidative stress; reversed by Genistein	Ameliorated Aβ toxicity; Neuroprotective against glucose
Bentivegna et al. (2025) *Aging Dis*	Amyloid Beta Regulates Astrocytic Glucose Metabolism and Insulin Signaling in Experimental Models of Alzheimer’s Disease	PDAPP-J20 mice / Primary astrocytes	Hyperinsulinemia, hippocampal IR	Aβ exposure induced oxidative stress in astrocytes	Aβ leads to alterations on insulin metabolism; insulin treatment improves mitochondrial dysfunction
Nagori et al. (2024) *Biocheml Biophys Res Comm*	Ethyl gallate ameliorates diabetes-induced Alzheimer’s disease-like phenotype in rats via activation of α7 nicotinic receptors and mitigation of oxidative stress	Rats (HFD + STZ)	Diabetes-induced AD phenotype	EG ¯ oxidative stress (Normalized GSH, SOD, CAT, LPO)	Ameliorated AD-like phenotype; Improved cognitive deficits
Akamba Ambamba et al. (2024) *J Ethnopharmacology*	Tannins-enriched fraction of TeMac™ protects against aluminum chloride induced Alzheimer’s disease-like pathology by modulating aberrant insulin resistance and alleviating oxidative stress in diabetic rats	Rats (AD and DM models AlCl₃+ STZ)	Induced DM	TEF of TeMac™ ¯ oxidative stress markers	Protected against AD-like pathology; ¯ neurotoxicity
Abosharaf *et al*. (2023) *J Neuroendocrinology*	Alzheimer’s disease‐related brain insulin resistance and the prospective therapeutic impact of metformin	Rats (STZ)	Brain IR	¯ Antioxidant defense ­ oxidative stress markers	Metformin attenuated memory loss, AD markers and oxidative stress
Tang et al. (2022) *Antioxid Redox Signal (ARS)*	Caveolin-1 Alleviates Diabetes-Associated Cognitive Dysfunction Through Modulating Neuronal Ferroptosis-Mediated Mitochondrial Homeostasis	Mice (HFD/STZ)	Diabetes-induced mitochondrial dysfunction.	Ferroptosis (oxidative iron-dependent cell death) modulation.	Ferroptosis promoted the progression of cognitive decline induced by T2D; Alleviated by Cav-1
Ansari et al. (2023) *Brain Res*	Early time course of oxidative stress in hippocampal synaptosomes and cognitive loss following impaired insulin signaling in rats: Development of sporadic Alzheimer’s disease	Rats (STZ)	Impaired insulin signaling	­hippocampal oxidative stress (¯ GSH; ­LPO)	Cognitive loss followed oxidative stress; Development of sporadic AD features
Akhtar et al. (2021) *Psychopharmacology*	7,8-Dihydroxyflavone improves cognitive functions in ICV-STZ rat model of sporadic Alzheimer’s disease by reversing oxidative stress, mitochondrial dysfunction, and insulin resistance	Rat (ICV-STZ)	Brain IR	¯ Antioxidant defense ­ oxidative stress markers and mitochondrial disfunction	7,8-DHF reversed oxidative stress and mitochondrial dysfunction; Improved cognitive functions
Shu et al. (2020) *Aging*	Loss of β-catenin via activated GSK3β causes diabetic retinal neurodegeneration by instigating a vicious cycle of oxidative stress-driven mitochondrial impairment	Mice (HFD) / Retinal tissue	Dysregulated insulin signaling	Oxidative stress (­4-HNE), mitochondrial impairment; ¯ antioxidant defense	Neurodegeneration linked to GSK3β/β-catenin pathway via ROS dysregulation
Ma et al. (2020) *Front Neurosci*	Neuroprotective Effect of Resveratrol via Activation of Sirt1 Signaling in a Rat Model of Combined Diabetes and Alzheimer’s Disease	Rat (STZ + Aβ injection)	Hyperglycemia	¯ SOD/GSH and ­ MDA	Resveratrol reversed oxidative stress; inhibited memory impairment
Elahi et al. (2016) *J Alzheimers Dis*	Region-Specific Vulnerability to Oxidative Stress, Neuroinflammation, and Tau Hyperphosphorylation in Experimental Diabetes Mellitus Mice	Mice (STZ)	Diabetes	­ ROS ­ LPO	Region-specific vulnerability relating neuroinflammation and oxidative stress to p-tau
Nuzzo et al. (2015) *Curr Alzheimer Res*	Insulin Resistance as Common Molecular Denominator Linking Obesity to Alzheimer’s Disease	Mice (HFD)	Obesity-induced Insulin Resistance	HFD ­oxidative stress and mitochondrial dysfunction	­ AD markers
Lester-Coll et al. (2006) *J Alzheimers Dis*	Intracerebral streptozotocin model of type 3 diabetes: Relevance to sporadic Alzheimer’s disease	Rat (ICV-STZ)	Type 3 diabetes (Brain insulin deficiency model)	­ Oxidative stress	Depletion of insulin and IGF signaling combined with oxidative damage cause AD-type neurodegeneration
Zagare et al. (2025) *J Tissue Eng*	Insulin resistance compromises midbrain organoid neuronal activity and metabolic efficiency predisposing to Parkinson’s disease pathology	Humans IPCs derived midbrain organoids	IR (Induced by high insulin concentration)	IR ­ Oxidative stress	¯ neuronal activity ; ¯ amount of dopaminergic neurons
Ramalingam & Kim (2014) *J Recept Signal Transduct Res*	The role of insulin against hydrogen peroxide-induced oxidative damages in differentiated SH-SY5Y cells	Cells (SH-SY5Y)	Insulin treatment	H_2_O_2_ ­damage; Insulin ¯ ROS	Insulin protected neuronal cells against oxidative damage
Duarte et al. (2006) *Diabetes*	Insulin restores metabolic function in cultured cortical neurons subjected to oxidative stress	Cells Rat cultured cortical neurons	Insulin treatment restored metabolic function	Oxidative stress ­ protein oxidation; ­ neuronal death; ¯ PI3K/AKT activation	Insulin prevented loss of metabolic function; Neuroprotective against oxidative injury
B) Oxidative stress and AD/neurodegeneration					
Ahmad et al. (2024) *Alzheimers Res Ther*	Association of oxidative stress and inflammatory metabolites with Alzheimer’s disease cerebrospinal fluid biomarkers in mild cognitive impairment	Humans (MCI patients)	N/A	Association between ­ Oxidative stress and ­ AD biomarkers (p-tau)	Oxidative stress and inflammation are linked to AD pathology in the MCI stage.
Sultana et al. (2011) *J Alzheimers Dis*	Increased protein and lipid oxidative damage in mitochondria isolated from lymphocytes from patients with Alzheimer’s disease	Humans (Lymphocytes: AD patients)	N/A	­ protein oxidations and LPO in mitochondria	Systemic mitochondrial oxidative stress in peripheral cells mirrors AD brain pathology.
Ansari & Scheff (2010) *J Neuropathol Exp Neurol*	Oxidative stress in the progression of Alzheimer disease in the frontal cortex	Humans (Post-mortem brains: MCI, AD)	N/A	­ Oxidative stress; ¯ antioxidant defense; Mitochondrial dysfunction	Oxidative stress is involved in AD synaptic loss.
Ramsey et al. (2007) *J Neuropathol Exp Neurol*	Expression of Nrf2 in Neurodegenerative Diseases	Humans (Post-mortem brains)	N/A	Altered Nrf2 expression	Nrf2 expression is altered in AD and other neurodegenerative diseases.
Cazzaro et al. (2023) *Proc Natl Acad Sci (PNAS)*	Slingshot homolog-1-mediated Nrf2 sequestration tips the balance from neuroprotection to neurodegeneration in Alzheimer’s disease	Mice / Cells (HT22 e HEK293T)	N/A	¯ Nrf2 leads to ¯ antioxidant defense, leading to oxidative damage	¯ Nrf2 activity drives neurodegeneration and AD pathology.
Rojo et al. (2017) *Redox Biol*	NRF2 deficiency replicates transcriptomic changes in Alzheimer’s patients and worsens APP and TAU pathology	Mice (NRF2 knockout / AD models)	N/A	Lack of NRF2 leads to oxidative stress.	NRF2 deficiency worsens AD pathology; replicates AD transcriptomic changes
Tamagno et al. (2007) *J Neurochem*	Oxidative stress activates a positive feedback between the γ- and β-secretase cleavages of the β-amyloid precursor protein	Cells/Mice SK-N-BE neuroblastoma and MEFs	N/A	Oxidative stress activates γ- andβ secretase enzymes.	Oxidative stress ­ Aβ production via BACE1
Su et al. (2010) *Neurosci Lett*	Chronic oxidative stress causes increased tau phosphorylation in M17 neuroblastoma cells	Cells M17 neuroblastoma	N/A	BSO induced oxidative stress	Chronic oxidative stress induced ­ p-tau
C) Oxidative stress and metabolic alterations (diabetes-related)					
Abu Khadra et al. (2024) *Eur J Med Res*	Oxidative stress and type 2 diabetes: the development and the pathogenesis, Jordanian cross-sectional study	Humans (Blood: T2D, obese patients and controls)	T2D status confirmed	­ MDA correlated with ¯ CAT levels in T2D and obese patients	N/A
Singh & Kumari (2021) *J Pharm Bioallied Sci*	Assessment of Correlation of Oxidative Stress and Insulin Resistance with Glucose-6-Phosphate Dehydrogenase Activity in Type II Diabetes Mellitus Patients	Humans (Blood: T2D patients and controls)	T2D confirmed; high HbA1C	¯ G6PD activity correlates with ­ oxidative stress and HbA1C.	N/A
Mahat et al. (2019) *Diabetes Metab Syndr*	Cross-sectional correlates of oxidative stress and inflammation with glucose intolerance in prediabetes	Humans (Pre-DM and controls)	Pre-DM status confirmed	­ Oxidative stress (MDA and 8-OHdG) and ¯ GSH in pre-DM patients.	N/A
Aouacheri et al. (2015) *Can J Diabetes*	The investigation of the oxidative stress-related parameters in type 2 diabetes mellitus	Humans (T2D patients and controls)	T2D status confirmed	­ Oxidative stress (MDA); ¯ antioxidant enzymes (CAT, GSH) in T2D patients.	N/A
Xavier et al. (2014) *Diabetes Res Clin Pract*	One-week intervention period led to improvements in glycemic control and reduction in DNA damage levels in patients with type 2 diabetes mellitus.	Humans (Blood - T2D patients)	Intervention improved glycemic control (HbA1c/Fasting Plasma Glucose).	Intervention ¯ DNA damage	N/A

The studies are listed based on model/population and year, starting from the most recent studies in humans, followed by in vivo animal models and *in vitro* studies. AD - Alzheimer’s disease; T2D - Type 2 Diabetes; DM: Diabetes Mellitus; IR: Insulin resistance; MCI - Mild Cognitive impairment; CMRglu: Cerebral Metabolic Rate of Glucose; HbA1c - glycated hemoglobin; HFD - High fat diet; STZ- streptozotocin; DrD - Diabetes related Dementia; CSF-Cerebral Spinal Fluid;VF - Ventricular fluid; AlCl₃- aluminum chloride; ICV - Intracerebroventricular; Oxidative stress markers (LPO - Lipid peroxidation ; MDA - Malondialdehyde; 8-OHdG - 8-hidroxi-2’-desoxiguanosina; 4-HNE - 4-hydroxynonenal); Antioxidant Enzymes (GSH - glutathione; SOD- Superoxide dismutase; CAT-Catalases).

## Oxidative stress and neurodegeneration

There is a body of evidence in the literature suggesting the association between oxidative stress and neurodegeneration (Table 2B). One of the reasons is that the brain is highly susceptible to oxidative damage due to its high oxygen consumption and elevated mitochondrial activity, thus triggering increased ROS production involved in redox signal transmission (Cobley et al., 2018). In addition, the brain is also rich in metal ions, which favors the Fenton reaction and thereby increases •OH formation (Todorich et al., 2009). Furthermore, neuronal membranes are rich in polyunsaturated fatty acids, which are particularly susceptible to lipid peroxidation (Bazinet and Layé, 2014). They also exhibit low levels of antioxidant enzymes, making them particularly vulnerable to oxidative stress-induced damage (Chen and Zhong, 2014; Ahmad et al., 2017).

Beyond the direct oxidative damage to cellular components, oxidative stress also disrupts intracellular signaling pathways that regulate glucose metabolism and neuronal survival. Because neuronal activity relies primarily on glucose oxidation for ATP production, any imbalance in redox homeostasis or metabolic regulation can severely compromise the brain, contributing to the development of several brain disorders (Traxler et al., 2021).

In this context, the insulin/PI3K/AKT signaling pathway is a central signaling axis that integrates oxidative and metabolic signals and regulates multiple cellular mechanisms, and alterations in its regulation can trigger a series of biological processes, leading to disease onset and progression. 

## Insulin/PI3K/AKT pathway

The Insulin/PI3K/AKT pathway is a complex network that is tightly regulated by redox balance. Moderate ROS levels maintain the PI3K/AKT pathway activated through the inhibition of the negative regulators of PI3K (phosphatidylinositol 3-kinase) and AKT: phosphatase and tensin homolog (PTEN) and protein phosphatase 2A (PP2A), respectively; conversely, under sustained oxidative stress, ROS activates the AMP-activated protein kinase (AMPK) signaling which inhibits AKT activity (Kma and Baruah, 2022).

**Figure 1 -:**
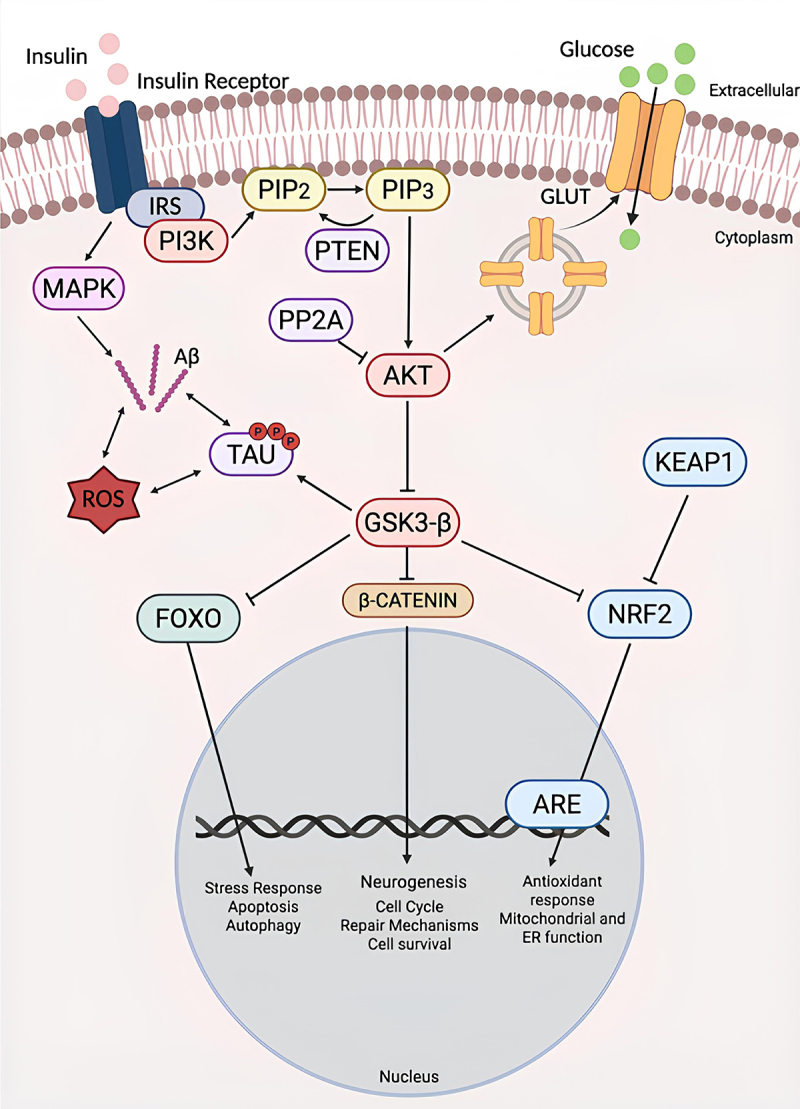
Insulin/PI3K/AKT pathway. Under physiological conditions, insulin binding to its receptor induces conversion of PIP2 into PIP3, activating PI3K and subsequently AKT, promoting glucose uptake through glucose transporter (GLUT) translocation. This process can be inhibited by PTEN, a negative regulator of the PI3K/AKT pathway. AKT inhibits GSK3-β, thereby sustaining NRF2 and FOXO activation and preventing β-catenin degradation. These effects enhance the stress and antioxidant responses, maintain mitochondrial and ER function, and support neurogenesis and cell survival. Inhibition of GSK3-β also prevents tau hyperphosphorylation and Aβ oligomer formation - key pathological hallmarks of Alzheimer’s disease. Aberrant MAPK expression and excessive ROS promote tau hyperphosphorylation and Aβ aggregation, exacerbating neuronal damage. ROS: Reactive oxygen species; ARE: Antioxidant Response Element; ER: Endoplasmic reticulum; NFTs: Neurofibrillary tangles; Aβ: amyloid beta. Figure created with BioRender.com.

Under normal physiological conditions, insulin and/or IGF-1 binds to insulin receptors or insulin-like growth factor receptors (IGF-1R), expressed both in peripheral target tissues, such as skeletal muscle, liver, and adipose tissue, and in the brain, particularly in neurons and glial cells, with higher density in the hippocampus, hypothalamus, cerebral cortex, and olfactory bulb (Kandimalla et al., 2016). Upon ligand binding, these receptors initiate a signaling cascade that activates PI3K. Subsequently, PI3K regulates the activity of AKT. AKT promotes the translocation of glucose transporters (GLUT) to the plasma membrane, thereby facilitating glucose uptake into the cells. In addition, through inhibition of FOXO transcription factors, AKT modulates the expression of genes involved in stress response, antioxidant defense, and autophagy (Kandimalla *et al*., 2016). AKT also phosphorylates and inhibits GSK-3β, modulating the antioxidant defense through NRF2 degradation, therefore AKT activation sustains NRF2 activation (Rada et al., 2011). GSK3-β is also involved in the hyperphosphorylation of tau protein and the formation of Aβ aggregates through the increase in amyloid precursor protein (APP) processing (Sims-Robinson et al., 2010) (Figure 1).

Insulin exerts multiple roles in neuronal function, including the regulation of neurite outgrowth and catecholaminergic neurotransmission, as well as the modulation of synaptic activity (Scherer et al., 2021). In neurons, insulin signaling is initiated by activation of the insulin receptor tyrosine kinase, leading to phosphorylation of insulin receptor substrates and downstream activation of the PI3K/AKT and MAPK/ERK pathways (Van der Heide *et al*., 2005). Through PI3K/AKT signaling, insulin modulates synaptic plasticity by regulating glutamatergic neurotransmission, involving trafficking and function of N-methyl-D-aspartate (NMDA) and α-amino-3-hydroxy-5-methyl-4-isoxazolepropionic acid (AMPA) receptors, thereby influencing long-term potentiation and depression of synapsis, particularly in the hippocampus (Van der Heide *et al*., 2005). Insulin also affects inhibitory transmission by modulating γ-aminobutyric acid (GABA) receptors activity and synaptic balance (Trujeque-Ramos et al., 2018). In addition, activation of AKT promotes neuronal survival by inhibiting apoptotic pathways, including phosphorylation of pro-apoptotic proteins such as Bad and suppression of caspase activation (Kim and Han, 2005). Moreover, activation of the insulin signaling pathway enhances neuronal glucose utilization during periods of increased metabolic demand, such as learning, and supports neuronal differentiation and survival (Brooker et al., 2000; Duarte et al., 2006; Pearson-Leary and McNay, 2016) (Figure 1).

It is important to highlight that phosphorylation within the insulin/PI3K/AKT pathway is crucial to glucose homeostasis and neuronal function. Insulin signaling also activates the mitogen-activated protein kinase (MAPK) pathway, which regulates cell differentiation, proliferation, and survival. Aberrant MAPK expression has been associated with Aβ plaques and NFTs in AD (Sims-Robinson et al., 2010). In T2D and AD, a low AKT activity results in GSK3-β activation, promoting oxidative stress, mitochondrial dysfunction, tau hyperphosphorylation and Aβ accumulation (Sims-Robinson *et al*., 2010; Shu et al., 2020). These findings provide a promising avenue for therapeutic strategies aimed at enhancing neuronal cell survival and neurogenesis in AD (Zheng et al., 2017). 

Overall, insulin signaling plays a pivotal role in cellular homeostasis, and conditions like insulin resistance significantly disrupt the insulin/PI3K/AKT pathway and trigger pathological changes.

## Insulin/PI3K/AKT connected to neurogenesis and neurodegeneration

Insulin and IGF-1 are key regulators of brain function, controlling cell proliferation, differentiation, and neural stem cell (NSC) survival (Brooker et al., 2000; Åberg et al., 2003). The NSCs located in specific niches, such as the subventricular zone (SVZ) and the subgranular zone (SGZ) of the dentate gyrus of the hippocampus, continue to produce new neurons during adulthood (Bond et al, 2015; Dioli et al, 2021). Adult neurogenesis, a form of brain plasticity that supports memory, relies on the tightly regulated activation of mostly quiescent NSCs within neurogenic niches, enabling neuronal and glial differentiation and circuit integration (Mu and Gage, 2011; Kempermann et al., 2018; Babcock et al., 2021). Disruption of hippocampal neurogenesis is implicated in AD, as long as this process declines with disease progression, but remains detectable at reduced levels in AD brains (Moreno-Jiménez et al., 2019; Tobin et al., 2019).

Activation of the insulin/PI3K/AKT pathway is essential for NSCs differentiation (Brooker et al., 2000). This occurs primarily via inhibition of GSK3-β, which, when active targets β-catenin for degradation. Conversely, GSK3-β inhibition allows β-catenin, nuclear translocation, and activation of genes such as *Cyclin-D* and *Neuro-D1*, thereby driving the differentiation of NSCs into neurons (Brooker *et al*., 2000; Liu et al., 2023) (Figure 1). However, chronic hyperactivation of GSK3-β has been found to inhibit antioxidant enzymes action, leading to oxidative stress, mitochondrial impairment, and neurodegeneration; consequently, it can also prematurely exhaust the neurogenic niche, compromising neuronal renewal (Sun, 2006; Shu et al., 2020). 

In this regard, impaired insulin sensitivity or signaling in the brain can directly affect synaptic plasticity and neurogenesis, thereby compromising cognitive functions and increasing vulnerability to neurodegenerative processes (Åberg et al., 2003; Elahi et al., 2016). Because peripheral and central insulin signaling are closely interconnected, metabolic disturbances in the body can directly impact brain function. Consequently, there is a growing prevalence of metabolic disorders in middle age, which occurs alongside an increase in cognitive decline (Gudala et al., 2013; Nuzzo et al., 2015). Progressive memory decline in AD is directly associated with hippocampal degeneration, affecting learning and memory, since the hippocampus is highly vulnerable to oxidative damage in the early stages of the disease (Mu and Gage, 2011; Lee et al., 2013; Babcock et al., 2021; Ansari et al., 2023). 

Together, these findings reinforce the conception that the normal functioning of insulin signaling is essential for neuronal survival, neurogenesis, and cognitive homeostasis, and its dysregulation constitutes a critical link between metabolic disorders and neurodegeneration.

## Protein metabolism dysregulation and neurodegeneration

Neurodegeneration can also be a consequence of oxidative modifications at the level of protein metabolism, making proteins susceptible to misfolding and aggregation. It is known that protein homeostasis is essential for neuronal survival and function, as it ensures the proper synthesis, folding, modification, and degradation of proteins. In glucose metabolism, the maintenance of cellular energy is highly dependent on a variety of proteins and key metabolic enzymes, and any alterations on these proteins can affect metabolic homeostasis, triggering a cascade of molecular events that can contribute to the development of many diseases, including T2D and neurodegenerative diseases, such as AD (Li et al., 2024; Zhout et al., 2025).

Protein post-translational modifications (PTMs), a regulatory mechanism involved in the reversible or irreversible addition or removal of covalent functional groups to amino acid side chains, play a crucial role in maintaining cellular homeostasis, mediating signal transduction, regulating metabolism and other essential cellular biological processes (Yang et al., 2023; Zhong et al., 2023). Among the several types of PTMs, (including phosphorylation, acetylation, ubiquitination, neddylation, O-GlcNAcylation, and methylation), ROS and RNS can also induce chemical modifications through oxidation and reduction (redox-PTMs). 

Redox-PTMs contribute to cellular signaling, by modulating downstream pathways and altering protein conformation, activity, and localization, thereby influencing cellular processes and redox homeostasis. However, accumulation of redox-PTMs, particularly throughout aging, can contribute to conformational changes that increase the propensity for protein misfolding and aggregation (Petrovic et al., 2021). In the brain, these alterations disturb proteostasis and impair neuronal function, processes that are critically involved in the onset and progression of neurodegenerative diseases.

In AD, oxidative stress-induced protein modifications can contribute to the abnormal processing of APP and tau, leading to the formation of Aβ plaques and NFTs. Under normal conditions, APP is cleaved by α-secretase and γ-secretases. However, in AD, APP undergoes abnormal processing by β- and γ-secretases. This amyloidogenic pathway generates insoluble Aβ peptides that aggregate into oligomers (Arbor et al., 2016). These oligomers accumulate and diffuse to synapses, affecting protein functionality and signaling, ultimately contributing to synaptic dysfunction and neurodegeneration (Griffiths and Grant, 2023). 

Moreover, it has been reported that oxidative stress enhances the amyloidogenic activity of β- and γ-secretases, inducing the accumulation of Aβ peptides (Tamagno et al., 2007). In turn, Aβ peptides promote mitochondrial dysfunction, increase ROS production, induce oxidative stress, and trigger tau hyperphosphorylation (Atamna and Boyle, 2006; Bassil et al., 2020). Therefore, under this condition, a vicious cycle is established between oxidative stress and Aβ accumulation, ultimately leading to neuronal death.

Oxidative stress has also been associated with the formation of NFTs through its effects on tau phosphorylation dynamics (Chu and Liu, 2018). Under physiological conditions, tau phosphorylation is tightly regulated by a balance between tau-directed kinases and phosphatases, ensuring proper tau-microtubule binding and stabilization. Chronic oxidative stress disrupts this balance by activating stress-responsive kinases, while simultaneously inhibiting PP2A, a key phosphatase involved in tau dephosphorylation, resulting in sustained tau hyperphosphorylation (Su et al., 2010). This aberrant hyperphosphorylation weakens tau-microtubule interaction, promotes tau self-aggregation, and facilitates the accumulation of intracellular NFTs, ultimately leading to microtubule destabilization (Chu and Liu, 2018). Collectively, these alterations indicate that chronic oxidative stress drives tau pathology, contributing to impaired axonal transport, synaptic dysfunction, mitochondrial and cytoskeletal alterations, and further exacerbation of oxidative stress, thereby culminating in neuronal death (Alavi Naini and Soussi-Yanicostas, 2015).

**Figure 2 -:**
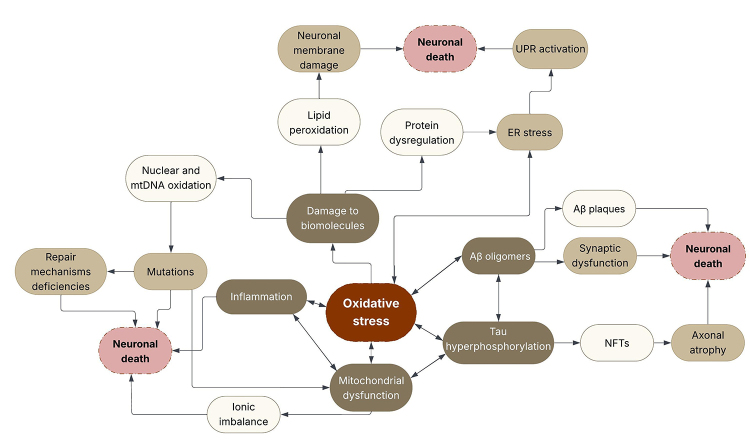
Schematic representation of alterations reported in several biological processes connected to oxidative stress, leading to neuronal death. Oxidative stress causes extensive damage to cellular biomolecules, including lipids, proteins, and DNA. Lipid peroxidation compromises neuronal membranes, while protein dysregulation contributes to endoplasmic reticulum (ER) stress and activation of the unfolded protein response (UPR). Oxidation of nuclear and mitochondrial DNA (mtDNA) promotes mutations and impairs repair mechanisms, further enhancing neuronal vulnerability. The pro-oxidant state and the induced damage may lead to neurodegeneration. In parallel, mitochondrial dysfunction and inflammation exacerbate reactive oxygen species (ROS) production, creating a cycle of oxidative damage. In Alzheimer’s disease (AD), oxidative stress also promotes amyloid beta (Aβ) aggregation and tau hyperphosphorylation, resulting in the formation of Aβ plaques and neurofibrillary tangles (NFTs). These molecular events culminate in synaptic dysfunction, axonal atrophy, and ultimately neuronal death, which are key hallmarks of neurodegenerative processes. (Modified from Ekundayo et al., 2024).

Therefore, as aforementioned, several biological processes can be compromised in consequence of chronic oxidative stress, leading to a series of metabolic and other pathological changes, ultimately culminating in neuronal death/neurodegeneration (as illustrated in Figure 2).

## ER stress and UPR activation

Another relevant mechanism through which oxidative stress disrupts protein homeostasis involves endoplasmic reticulum (ER) stress. While the ER normally adapts its folding capacity to preserve cellular homeostasis, sustained oxidative stress promotes the accumulation of misfolded proteins, overwhelming ER capacity and triggering ER stress and activation of the unfolded protein response (UPR) (Ekundayo et al., 2024).

**Figure 3 -:**
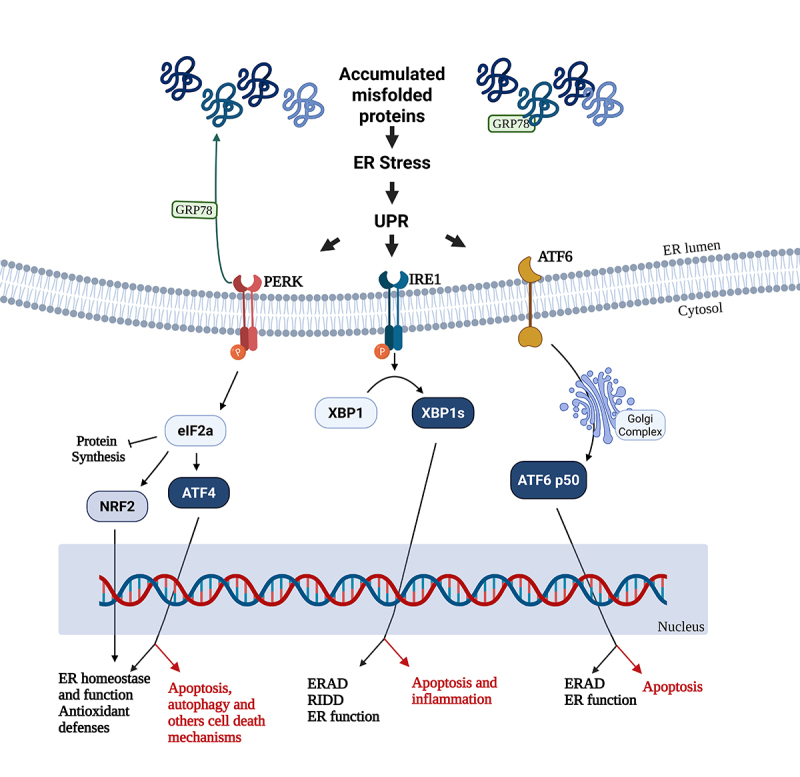
Unfolded protein response (UPR) cascade. The accumulation of misfolded proteins induces endoplasmic reticulum (ER) stress, activating the UPR sensors PERK, IRE1 and ATF6. These sensors activate adaptive responses through specific transcription factors that upregulate genes involved in various processes: protein synthesis, ER capacity, chaperone production, autophagy, and antioxidant defenses (black arrows). Sustained ER stress and UPR activation can shift toward a terminal response, ultimately triggering cell death pathways (red arrows). Figure created with BioRender.com.

The ER is a multifunctional and interconnected organelle involved in protein assembly/folding and post-translational modifications, calcium homeostasis, lipid biosynthesis, metabolic regulation, as well as in the regulation of apoptosis and mitochondrial performance (Ekundayo et al., 2024). These processes are highly sensitive to redox imbalance, and their disruption under sustained oxidative stress compromises ER function. In response to ER stress, cells activate the UPR, an adaptive signaling mechanism mediated by three main ER stress sensors: protein kinase-like ER kinase (PERK), inositol-requiring transmembrane kinase/endoribonuclease 1α (IRE1α), and activating transcription factor 6 (ATF6), which are normally kept inactive through binding to the chaperone BiP/GRP78 (Ajoolabady et al., 2022). Upon stress, BiP/GRP78 dissociates from these sensors, initiating signaling cascades that attenuate global protein synthesis, activate antioxidant responses, and enhance ER folding capacity in an attempt to restore ER homeostasis and function (Iurlaro and Muñoz-Pinedo, 2016) (Figure 3).

When ER stress becomes excessive, prolonged, or persistent, UPR signaling shifts from an adaptive to a maladaptive response, culminating in the activation of cell death pathways (Ajoolabady et al., 2022). Sustained PERK/ATF4 signaling induces the expression of pro-apoptotic genes and genes associated with autophagy (Iurlaro and Muñoz-Pinedo, 2016; Yao et al., 2017). In parallel, chronic activation of the IRE1α/XBP1 pathway can trigger apoptotic and pro-inflammatory signaling through stress kinases and inflammatory mediators (Shi et al., 2022; Hemagirri et al., 2024) (Figure 3). ATF6 activation further contributes to these responses by regulating genes and proteins involved in ER-associated degradation and protein folding; however, under sustained stress, ATF6 also promotes neuronal death by apoptosis (Ajoolabady *et al*., 2022; Wodrich et al., 2022). Collectively, sustained ER stress and maladaptive UPR activation can trigger multiple regulated cell death pathways, including apoptosis, necroptosis, ferroptosis and pyroptosis (reviewed in Shi *et al*., 2022) (Figure 3).

In AD, the persistent ER stress, primarily driven by calcium imbalance, Aβ and NFTs, has been shown to contribute to neuronal dysfunction and disease progression (Shi et al., 2022; Hemagirri et al., 2024). Although studies using human subjects and cellular models investigating the effects and mechanisms of UPR in AD are still scarce and limited, emerging data support a relevant role for ER stress-related pathways in neurodegenerative processes. 

In this context, Aβ₁-₄₂ injection in rat brains increases ER stress markers (GRP78, GADD153), activates ER-associated caspases-12 and -3, reduces antioxidant proteins, and induces neuronal damage leading to neurodegeneration (Goswami et al., 2020). Post-mortem AD brain analyses corroborate the involvement of ER stress and UPR dysregulation, particularly in hippocampal neurons, characterized by the co-accumulation of phosphorylated PERK (p-PERK) and tau, along with aberrant GSK3-β activation, linking ER stress to core AD pathologies (Hoozemans et al., 2009). p-PERK levels are markedly elevated in the AD cortex and positively correlate with Braak stage, a semiquantitative measure of NFT progression (Buchanan et al., 2020). Consistently, impaired ER proteostasis is evidenced by reduced GRP78 expression in PS1-mutant human cell lines and in sporadic and familial AD brains, as well as by pathological post-translational modifications of protein disulfide isomerase (PDI), contributing to ER dysfunction (Katayama et al., 1999).

Beyond these findings, ER stress and UPR signaling are also thought to contribute to the increased risk of AD in patients with T2D. Chronic hyperglycemia and insulin resistance have been shown to induce ER stress and sustained UPR activation, leading to pancreatic β-cell exhaustion and apoptosis (Chen et al., 2022). Furthermore, the overload provoked by hyperglycemia increases the production of AGEs and activates RAGE, which impairs Aβ clearance and stimulates neuroinflammation and oxidative stress (Cheng et al., 2025). In this scenario, metabolic disturbances associated with T2D may exacerbate ER stress-related pathways in the brain, thereby accelerating and aggravating AD pathology.

Together, the evidence discussed supports the assumption that chronic oxidative stress-induced ER dysfunction represents a critical interface between metabolic dysregulation and neurodegeneration, providing a mechanistic insight into how T2D-related disturbances may increase the risk of developing AD later in life. 

## Challenges and future perspectives

### Models for neurodegenerative research

Understanding how oxidative stress and metabolic imbalance drive molecular and cellular alterations that contribute to neurodegenerative diseases remains a major challenge. Accordingly, the development of experimental models that better capture these processes in human neural systems has become an important research topic, but still presents a major limitation regarding the use of suitable neural models. 

Several studies have been conducted on cell lines transformed from tumor tissues, such as SH-SY5Y or PC12 cells, due to characteristics such as accessibility, scalability, ease of genetic manipulation, and reproducibility, their use provides valuable mechanistic insights under more controlled and reductionist conditions (Cetin et al., 2022; Moreira et al., 2022, 2025; Ono et al., 2025). However, their tumor-derived or non-neuronal origins confer altered signaling pathways and incomplete neuronal identity (Forster et al., 2016). Consequently, the development of in vitro neural models derived from non-neoplastic human cells has emerged, with significant progress achieved over the last decade.

Primary neuronal cultures, often derived from neonatal rodent brains, preserve key features of in vivo neurons and have been valuable for mechanistic and pharmacological studies, allowing detailed investigations into cellular mechanisms and drug efficacy (Zhang et al., 2022 b ; Rozumna et al., 2023). Additionally, animal models have been widely used to probe the mechanistic links between metabolic dysfunction, cerebral insulin resistance, and neurodegenerative pathology (Ma et al., 2020; Nagori et al., 2024). These systems have demonstrated that metabolic stress can exacerbate disease-relevant features, including AD-like alterations (Elahi et al., 2016; Sankar et al., 2020). Nevertheless, their translation fidelity remains limited, particularly for studying chronic oxidative stress, metabolic alterations, and aging-related processes (Zhang *et al*., 2022b). Substantial interspecies differences in brain metabolism, redox regulation, and Aβ and tau biology undermine the predictive power of these models and have contributed to repeated failures in clinical translation (Granzotto et al., 2024). 

In this context, human induced pluripotent stem cell (iPSCs)-derived neural cultures advance the field by enabling the study of patient genetic backgrounds and disease variants with higher fidelity (Ochalek et al., 2017). Still, the reprogramming process resets aging and epigenetic signatures, limiting their ability to capture key aspects of metabolic and neurodegenerative diseases (Valdes et al., 2023). To address this limitation, direct conversion of patient fibroblasts into induced neurons (iNs) preserves aging-related epigenetic landscapes and cellular damage (Mertens et al., 2015; Sun et al., 2024). This feature is particularly relevant for studying oxidative stress and metabolic alterations that emerge with aging and contribute to neurodegeneration. Nevertheless, these 2D systems lack the cytoarchitectural complexity and cellular diversity characteristic of the human brain, limiting their capacity to recapitulate disease phenotypes. 

Three-dimensional (3D) models capture aspects of brain architecture and cell-cell interactions that are absent in 2D systems. Neural spheroids, generated through the aggregation of neural progenitor cells, allow the study of intercellular communication and exhibit improved survival, maturation, and intercellular communication (Park et al., 2023; Strong et al., 2023), but remain limited in cellular diversity and structural organization (Strong *et al*., 2023). In contrast, iPSC-derived brain organoids generate self-organized structures that recapitulate key features of human cortical development, including cytoarchitecture, cellular diversity, and long-term maturation (Trujillo et al., 2019; Vanova et al., 2023). These platforms provide a valuable opportunity to investigate how metabolic stress and redox imbalance affect neuronal function and survival in a human-specific context and have already been used to study insulin signaling pathways in neurodegenerative diseases, revealing how metabolic stress can exacerbate age-related disorders in the brain (Zagare et al., 2025).

### Future perspectives: Multi-Omic strategies and advanced human neural models

Despite the substantial body of evidence linking oxidative stress, metabolic dysfunction, and neurodegeneration, several mechanistic aspects underlying the connection between T2D and AD remain to be clarified. Addressing these gaps may require integrative strategies capable of elucidating the complex molecular networks that connect both diseases.

Although transcriptomic analyses have revealed shared molecular alterations between AD and T2D, gene expression profiles do not necessarily reflect functional protein changes. In this context, the expansion of high-throughput proteomic approaches represents an important current step, although the identification of molecular targets through these methods remains a major challenge. Notably, studies based on well-characterized patient cohorts (for example, individuals presenting both T2D and AD) are still limited due to difficulties in patient stratification and longitudinal follow-up. The lack of such cohorts restricts deeper insights into how metabolic dysfunction progresses to neurodegenerative processes in the brain. Advancing multi-omic approaches in carefully stratified populations may help clarify shared mechanisms, possibly enabling translational applications in the future.

Regarding studies using human brain tissue, large-scale proteomic studies face additional challenges and are limited by sample accessibility, post-mortem variability, and disease heterogeneity. Combining transcriptomics, proteomics, and metabolomics in peripheral samples, human brain tissue, and advanced human neural models provide expectations towards achieving a more comprehensive understanding of disease progression and mechanistic knowledge regarding the connection between T2D and AD.

Furthermore, the continued development of human-based neural models (including iNs, 3D organoids, and assembloids) offers valuable opportunities to investigate disease mechanisms within physiologically relevant and patient-specific contexts. These platforms enable the integration of metabolic stress, aging signatures, vascular components, and neuroinflammatory processes, thereby facilitating the identification of early molecular events linking T2D to AD. Together with multi-omic data, such models may allow the development of predictive biomarkers and the identification of potential molecular targets that may be applied in therapeutic strategies.

From a therapeutic perspective, oxidative stress remains a central target in both metabolic and neurodegenerative diseases. Although antioxidant-based interventions and NRF2 activation strategies have shown neuroprotective and metabolic benefits in experimental and clinical contexts (Asbaghi et al., 2023; Sharkus et al., 2023; Sun et al., 2023), future efforts should move beyond single-target approaches. Given the complex interplay between insulin signaling, mitochondrial dysfunction, ER stress, protein dysregulation, and redox imbalance, multi-target therapeutic approaches that address multiple converging pathways, combined with personalized medicine approaches (which consider each patient as a unique context), may offer more effective interventions for both conditions.

## Data Availability

No new data was created in this work.

## References

[B1] Åberg MAI, Åberg ND, Palmer TD, Alborn AM, Carlsson-Skwirut C, Bang P, Rosengren LE, Olsson T, Gage FH, Eriksson PS (2003). IGF-I has a direct proliferative effect in adult hippocampal
progenitor cells. Mol Cell Neurosci.

[B2] Abosharaf HA, Elsonbaty Y, Tousson E, M Mohamed T (2024). Alzheimer’s disease-related brain insulin resistance and the
prospective therapeutic impact of metformin. J Neuroendocrinol.

[B3] Abu Khadra KM, Bataineh MI, Khalil A, Saleh J (2024). Oxidative stress and type 2 diabetes: the development and the
pathogenesis, Jordanian cross-sectional study. Eur J Med Res.

[B4] Ahmad S, Yang W, Orellana A, Frölich L, de Rojas I, Cano A, Boada M, Hernández I, Hausner L, Harms AC (2024). Association of oxidative stress and inflammatory metabolites with
Alzheimer’s disease cerebrospinal fluid biomarkers in mild cognitive
impairment. Alzheimers Res Ther.

[B5] Ahmad W, Ijaz B, Shabbiri K, Ahmed F, Rehman S (2017). Oxidative toxicity in diabetes and Alzheimer’s disease:
Mechanisms behind ROS/RNS generation. J Biomed Sci.

[B6] Alzheimer’s Disease International (2022). From Plan to Impact V: WHO Global action plan: The time to act is
now. Alzheimer’s Dis Int.

[B7] Ajoolabady A, Lindholm D, Ren J, Pratico D (2022). ER stress and UPR in Alzheimer’s disease: Mechanisms,
pathogenesis, treatments. Cell Death Dis.

[B8] Akamba Ambamba BD, Ella FA, Ngassa Ngoumen DJ, Dibacto Kemadjou RE, Agwe NI, Mbappe FE, Fonkoua M, Enyegue DM, Ngondi JL (2024). Tannins-enriched fraction of TeMac™ protects against aluminum
chloride induced Alzheimer’s disease-like pathology by modulating aberrant
insulin resistance and alleviating oxidative stress in diabetic
rats. J Ethnopharmacol.

[B9] Akhtar A, Dhaliwal J, Sah SP (2021). 7,8-Dihydroxyflavone improves cognitive functions in ICV-STZ rat
model of sporadic Alzheimer’s disease by reversing oxidative stress,
mitochondrial dysfunction, and insulin resistance. Psychopharmacology (Berl).

[B10] Al-Aubaidy HA, Jelinek HF (2011). Oxidative DNA damage and obesity in type 2 diabetes
mellitus. Eur J Endocrinol.

[B11] Alavi Naini SM, Soussi-Yanicostas N (2015). Tau hyperphosphorylation and oxidative stress, a critical vicious
circle in neurodegenerative tauopathies?. Oxid Med Cell Longev.

[B12] An Y, Varma VR, Varma S, Casanova R, Dammer E, Pletnikova O, Chia CW, Egan JM, Ferrucci L, Troncoso J (2017). Evidence for brain glucose dysregulation in Alzheimer’s
disease. Alzheimers Dement.

[B13] Anderson AP, Luo X, Russell W, Yin YW (2020). Oxidative damage diminishes mitochondrial DNA polymerase
replication fidelity. Nucleic Acids Res.

[B14] Ansari MA, Scheff SW (2010). Oxidative stress in the progression of Alzheimer disease in the
frontal cortex. J Neuropathol Exp Neurol.

[B15] Ansari MA, Rao MS, Al-Jarallah A, Babiker FM (2023). Early time course of oxidative stress in hippocampal synaptosomes
and cognitive loss following impaired insulin signaling in rats: Development
of sporadic Alzheimer’ s disease. Brain Res.

[B16] Aouacheri O, Saka S, Krim M, Messaadia A, Maidi I (2015). The investigation of the oxidative stress-related parameters in
type 2 diabetes mellitus. Can J Diabetes.

[B17] Arbor SC, Lafontaine M, Cumbay M (2016). Amyloid-beta Alzheimer targets - protein processing, lipid rafts,
and amyloid-beta pores. Yale J Biol Med.

[B18] Asbaghi O, Nazarian B, Yousefi M, Anjom-Shoae J, Rasekhi H, Sadeghi O (2023). Effect of vitamin E intake on glycemic control and insulin
resistance in diabetic patients: An updated systematic review and
meta-analysis of randomized controlled trials. Nutr J.

[B19] Atamna H, Boyle K (2006). Amyloid-β peptide binds with heme to form a peroxidase:
Relationship to the cytopathologies of Alzheimer’s disease. Proc Natl Acad Sci U S A.

[B20] Averill-Bates D (2024). Reactive oxygen species and cell signaling.
Review. Biochim Biophys Acta Mol Cell Res.

[B21] Babcock KR, Page JS, Fallon JR, Webb AE (2021). Adult hippocampal neurogenesis in aging and Alzheimer’s
disease. Stem Cell Reports.

[B22] Baker LD, Cross DJ, Minoshima S, Belongia D, Stennis Watson G, Craft S (2011). Insulin resistance and Alzheimer-like reductions in regional
cerebral glucose metabolism for cognitively normal adults with prediabetes
or early type 2 diabetes. Arch Neurol.

[B23] Barbiellini AC, Fayosse A, Dumurgier J, Machado-Fragua MD, Tabak AG, van Sloten T, Kivimäki M, Dugravot A, Sabia S, Singh-Manoux A (2021). Association between age at diabetes onset and subsequent risk of
dementia. JAMA.

[B24] Bassil F, Brown HJ, Pattabhiraman S, Iwasyk JE, Maghames CM, Meymand ES, Cox TO, Riddle DM, Zhang B, Trojanowski JQ (2020). Amyloid-Beta (Aβ) plaques promote seeding and spreading of
alpha-synuclein and tau in a mouse model of lewy body disorders with aβ
pathology. Neuron.

[B25] Bazinet RP, Layé S (2014). Polyunsaturated fatty acids and their metabolites in brain
function and disease. Nat Rev Neurosci.

[B26] Bentivegna M, Pomilio C, Bellotto M, Pérez NG, Rossi SP, Gregosa A, Vota D, Merech F, Bonaventura MM, Presa J (2025). Amyloid beta regulates astrocytic glucose metabolism and insulin
signaling in experimental models of Alzheimer’s disease. Aging Dis.

[B27] Bhatia V, Sharma S (2021). Role of mitochondrial dysfunction, oxidative stress and autophagy
in progression of Alzheimer’s disease. J Neurol Sci.

[B28] Blasiak J, Arabski M, Krupa R, Wozniak K, Zadrozny M, Kasznicki J, Zurawska M, Drzewoski J (2004). DNA damage and repair in type 2 diabetes mellitus. Mutat Res Mol Mech Mutagen.

[B29] Bond AM, Ming GL, Song H (2015). Adult mammalian neural stem cells and neurogenesis: Five decades
later. Cell Stem Cell.

[B30] Brieger K, Schiavone S, Miller FJ, Krause KH (2012). Reactive oxygen species: From health to disease. Swiss Med Wkly.

[B31] Brooker GJ., Kalloniatis M, Russo VC, Murphy M, Werther GA, Bartlett PF (2000). Endogenous IGF-1 regulates the neuronal differentiation of adult
stem cells. J Neurosci Res.

[B32] Buchanan H, Mackay M, Palmer K, Tothová K, Katsur M, Platt B, Koss DJ (2020). Synaptic Loss, ER Stress and neuro-inflammation emerge late in
the lateral temporal cortex and associate with progressive tau pathology in
Alzheimer’s disease. Mol Neurobiol.

[B33] Byun K, Yoo YC, Son M, Lee J, Jeong GB, Park YM, Salekdeh GH, Lee B (2017). Advanced glycation end-products produced systemically and by
macrophages: A common contributor to inflammation and degenerative
diseases. Pharmacol Ther.

[B34] Cazzaro S, Woo JAA, Wang X, Liu T, Rego S, Kee TR, Koh Y, Vázquez-Rosa E, Pieper AA, Kang DE (2023). Slingshot homolog-1-mediated Nrf2 sequestration tips the balance
from neuroprotection to neurodegeneration in Alzheimer’s
disease. Proc Natl Acad Sci U S A.

[B35] Cetin S, Knez D, Gobec S, Kos J, Pišlar A (2022). Cell models for Alzheimer’s and Parkinson’s disease: At the
interface of biology and drug discovery. Biomed Pharmacother.

[B36] Chen CW, Guan BJ, Alzahrani MR, Gao Z, Gao L, Bracey S, Wu J, Mbow CA, Jobava R, Haataja L (2022). Adaptation to chronic ER stress enforces pancreatic β-cell
plasticity. Nat Commun.

[B37] Chen TN, Orr AL, Orr AG, Vacanti NM (2025). Identifying links between Alzheimer’s disease and Type 2 diabetes
through proteomics. J Proteome Res.

[B38] Chen Z, Zhong C (2013). Decoding Alzheimer’s disease from perturbed cerebral glucose
metabolism: Implications for diagnostic and therapeutic
strategies. Prog Neurobiol.

[B39] Chen Z, Zhong C (2014). Oxidative stress in Alzheimer’s disease. Neurosci Bull.

[B40] Cheng S, Xiao B, Luo Z (2025). Glycosylation in neuroinflammation: Mechanisms, implications, and
therapeutic strategies for neurodegenerative diseases. Transl Neurodegener.

[B41] Chu D, Liu F (2018). Pathological changes of tau related to Alzheimer’s
Disease. ACS Chem Neurosci.

[B42] Cobley JN, Fiorello ML, Bailey DM (2018). 13 reasons why the brain is susceptible to oxidative
stress. Redox Biol.

[B43] Cuadrado A, Kügler S, Lastres-Becker I (2018). Pharmacological targeting of GSK-3 and NRF2 provides
neuroprotection in a preclinical model of tauopathy. Redox Biol.

[B44] de Leon MJ, Ferris SH, George AE, Christman DR, Fowler JS, Gentes C, Reisberg B, Gee B, Emmerich M, Yonekura Y (1983). Positron emission tomographic studies of aging and Alzheimer
disease. AJNR Am J Neuroradiol.

[B45] Dioli C, Patrício P, Pinto LG, Marie C, Morais M, Vyas S, Bessa JM, Pinto L, Sotiropoulos I (2021). Adult neurogenic process in the subventricular zone‐olfactory
bulb system is regulated by Tau protein under prolonged
stress. Cell Prolif.

[B46] Duarte AI, Proença T, Oliveira CR, Santos MS, Rego AC (2006). Insulin restores metabolic function in cultured cortical neurons
subjected to oxidative stress. Diabetes.

[B47] Ekundayo BE, Obafemi TO, Adewale OB, Obafemi BA, Oyinloye BE, Ekundayo SK (2024). Oxidative Stress, endoplasmic reticulum stress and apoptosis in
the pathology of Alzheimer’s disease. Cell Biochem Biophys.

[B48] Elahi M, Hasan Z, Motoi Y, Matsumoto SE, Ishiguro K, Hattori N (2016). Region-specific vulnerability to oxidative stress,
neuroinflammation, and tau hyperphosphorylation in experimental diabetes
mellitus mice. J Alzheimer’s Dis.

[B49] Fão L, Mota SI, Rego AC (2019). Shaping the Nrf2-ARE-related pathways in Alzheimer’s and
Parkinson’s diseases. Ageing Res Rev.

[B50] Forster JI, Köglsberger S, Trefois C, Boyd O, Baumuratov AS, Buck L, Balling R, Antony PMA (2016). Characterization of differentiated SH-SY5Y as neuronal screening
model reveals increased oxidative vulnerability. SLAS Discov.

[B51] Gasparotto J, Girardi CS, Somensi N, Ribeiro CT, Moreira JCF, Michels M, Sonai B, Rocha M, Steckert AV, Barichello T (2018). Receptor for advanced glycation end products mediates
sepsis-triggered amyloid-β accumulation, Tau phosphorylation, and cognitive
impairment. J Biol Chem.

[B52] Goswami P, Afjal MA, Akhter J, Mangla A, Khan J, Parvez S, Raisuddin S (2020). Involvement of endoplasmic reticulum stress in amyloid β
(1-42)-induced Alzheimer’s like neuropathological process in rat
brain. Brain Res Bull.

[B53] Granzotto A, Vissel B, Sensi SL (2024). Lost in translation: Inconvenient truths on the utility of mouse
models in Alzheimer’s disease research. Elife.

[B54] Griffiths J, Grant SGN (2023). Synapse pathology in Alzheimer’s disease. Semin Cell Dev Biol.

[B55] Gudala K, Bansal D, Schifano F, Bhansali A (2013). Diabetes mellitus and risk of dementia: A meta‐analysis of
prospective observational studies. J Diabetes Investig.

[B56] Hatanaka H, Hanyu H, Fukasawa R, Sato T, Shimizu S, Sakurai H (2016). Peripheral oxidative stress markers in diabetes-related
dementia. Geriatr Gerontol Int.

[B57] Hemagirri M, Chen Y, Gopinath SCB, Sahreen S, Adnan M, Sasidharan S (2024). Crosstalk between protein misfolding and endoplasmic reticulum
stress during ageing and their role in age-related disorders. Biochimie.

[B58] Hokama M, Oka S, Leon J, Ninomiya T, Honda H, Sasaki K, Iwaki T, Ohara T, Sasaki T, Fm LaFerla (2014). Altered expression of diabetes-related genes in Alzheimer’s
disease brains: The Hisayama study. Cereb Cortex.

[B59] Hoozemans JJM, Van Haastert ES, Nijholt DAT, Rozemuller AJM, Eikelenboom P, Scheper W (2009). The unfolded protein response is activated in pretangle neurons
in alzheimer’s disease hippocampus. Am J Pathol.

[B60] Hoyos CM, Colagiuri S, Turner A, Ireland C, Naismith SL, Duffy SL (2022). Brain oxidative stress and cognitive function in older adults
with diabetes and pre-diabetes who are at risk for dementia. Diabetes Res Clin Pract.

[B61] International Diabetes Federation (2025). IDF Diabetes Atlas.

[B62] Iurlaro R, Muñoz-Pinedo C (2016). Cell death induced by endoplasmic reticulum
stress. FEBS J.

[B63] James DE, Stöckli J, Birnbaum MJ (2021). The aetiology and molecular landscape of insulin
resistance. Nat Rev Mol Cell Biol.

[B64] Jiang T, Zhang Y, Dai F, Liu C, Hu H, Zhang Q (2022). Advanced glycation end products and diabetes and other metabolic
indicators. Diabetol Metab Syndr.

[B65] Jolivalt CG, Lee CA, Beiswenger KK, Smith JL, Orlov M, Torrance MA, Masliah E (2008). Defective insulin signaling pathway and increased GSK-3 activity
in the brain of diabetic mice: Parallels with Alzheimer’s disease and
correction by insulin. J Neurosci Res.

[B66] Kandimalla R, Thirumala V, Reddy PH (2016). Is Alzheimer’s disease a type 3 diabetes? A critical
appraisal. Biochim Biophys Acta.

[B67] Katayama T, Imaizumi K, Sato N, Miyoshi K, Kudo T, Hitomi J, Morihara T, Yoneda T, Gomi F, Mori Y (1999). Presenilin-1 mutations downregulate the signalling pathway of the
unfolded-protein response. Nat Cell Biol.

[B68] Kempermann G, Gage FH, Aigner L, Song H, Curtis MA, Thuret S, Kuhn HG, Jessberger S, Frankland PW, Cameron HA (2018). Human adult neurogenesis: evidence and remaining
questions. Cell Stem Cell.

[B69] Kim SJ, Han Y (2005). Insulin inhibits AMPA-induced neuronal damage via stimulation of
protein kinase B (Akt). J Neural Transm.

[B70] Kma L, Baruah TJ (2022). The interplay of ROS and the PI3K/Akt pathway in autophagy
regulation. Biotechnol Appl Biochem.

[B71] Lee S, Tong M, Hang S, Deochand C, de la Monte S (2013). CSF and brain indices of insulin resistance, oxidative stress and
neuro-inflammation in early versus late Alzheimer’s disease. J Alzheimer’s Dis Park.

[B72] Lee SH, Park SY, Choi CS (2021). Insulin resistance: From mechanisms to therapeutic
strategies. Diabetes Metab J.

[B73] Lester-Coll N, Rivera EJ, Soscia SJ, Doiron K, Wands JR, de la Monte SM (2006). Intracerebral streptozotocin model of type 3 diabetes: Relevance
to sporadic Alzheimer’s disease. J Alzheimer’s Dis.

[B74] Li W, Li HL, Wang JZ, Liu R, Wang X (2024). Abnormal protein post-translational modifications induces
aggregation and abnormal deposition of protein, mediating neurodegenerative
diseases. Cell Biosci.

[B75] Lima JEBF, Moreira NCS, Sakamoto-Hojo ET (2022). Mechanisms underlying the pathophysiology of type 2 diabetes:
From risk factors to oxidative stress, metabolic dysfunction, and
hyperglycemia. Mutat Res Genet Toxicol Environ Mutagen.

[B76] Liu Q, Telezhkin V, Jiang W, Gu Y, Wang Y, Hong W, Tian W, Yarova P, Zhang G, Lee SM (2023). Electric field stimulation boosts neuronal differentiation of
neural stem cells for spinal cord injury treatment via
PI3K/Akt/GSK-3β/β-catenin activation. Cell Biosci.

[B77] Lowell BB, Shulman GI (2005). Mitochondrial dysfunction and type diabetes. Science.

[B78] Ma XR, Sun ZK, Han X, Li S, Jiang X, Chen S, Zhang J, Lu H (2020). Neuroprotective effect of resveratrol via activation of sirt1
signaling in a rat model of combined diabetes and Alzheimer’s
disease. Front Neurosci.

[B79] Mahat RK, Singh N, Rathore V, Arora M, Yadav T (2019). Cross-sectional correlates of oxidative stress and inflammation
with glucose intolerance in prediabetes. Diabetes Metab Syndr.

[B80] Malik AN, Parsade CK, Ajaz S, Crosby-Nwaobi R, Gnudi L, Czajka A, Sivaprasad S (2015). Altered circulating mitochondrial DNA and increased inflammation
in patients with diabetic retinopathy. Diabetes Res Clin Pract.

[B81] Mertens J, Paquola ACM, Ku M, Hatch E, Böhnke L, Ladjevardi S, McGrath S, Campbell B, Lee H, Herdy JR (2015). Directly reprogrammed human neurons retain aging-associated
transcriptomic signatures and reveal age-related nucleocytoplasmic
defects. Cell Stem Cell.

[B82] Moreira NCDS, Lima JEBF, Marchiori MF, Carvalho I, Sakamoto-Hojo ET (2022). Neuroprotective effects of cholinesterase inhibitors: Current
scenario in therapies for Alzheimer’s disease and future
perspectives. J Alzheimer’s Dis Reports.

[B83] Moreira NCDS, Piassi LO, Lima JEBF, Passos GA, Sakamoto-Hojo ET (2025). PTEN inhibition induces neuronal differentiation and
neuritogenesis in SH-SY5Y cells via AKT signaling pathway. J Alzheimer’s Dis.

[B84] Moreno-Jiménez EP, Flor-García M, Terreros-Roncal J, Rábano A, Cafini F, Pallas-Bazarra N, Ávila J, Llorens-Martín M (2019). Adult hippocampal neurogenesis is abundant in neurologically
healthy subjects and drops sharply in patients with Alzheimer’s
disease. Nat Med.

[B85] Mosconi L (2005). Brain glucose metabolism in the early and specific diagnosis of
Alzheimer’s disease: FDG-PET studies in MCI and AD. Eur J Nucl Med Mol Imaging.

[B86] Mu Y, Gage FH (2011). Adult hippocampal neurogenesis and its role in Alzheimer’s
disease. Mol Neurodegener.

[B87] Nagori K, Pradhan M, Nakhate KT (2024). Ethyl gallate ameliorates diabetes-induced Alzheimer’ s
disease-like phenotype in rats via activation of α 7 nicotinic receptors and
mitigation of oxidative stress. Biochem Biophys Res Commun.

[B88] Nuzzo D, Picone P, Baldassano S, Caruana L, Messina E, Gammazza A, Cappello F, Mulè F, Carlo M (2015). Insulin resistance as common molecular denominator linking
obesity to Alzheimer’s disease. Curr Alzheimer Res.

[B89] Ochalek A, Mihalik B, Avci HX, Chandrasekaran A, Téglási A, Bock I, Giudice ML, Táncos Z, Molnár K, László L (2017). Neurons derived from sporadic Alzheimer’s disease iPSCs reveal
elevated TAU hyperphosphorylation, increased amyloid levels, and GSK3B
activation. Alzheimers Res Ther.

[B90] Ono RMS, Moreira NCS, Carvalho I, Passos GA, Sakamoto-Hojo ET (2025). Novel donepezil-tacrine hybrid (TAHB3) induces
neurodifferentiation, neuroprotective effects, and activates the PI3K/AKT
pathway on PC12 cells. J Alzheimer’s Dis Reports.

[B91] Ott A, Stolk RP, Van Harskamp F, Pols HAP, Hofman A, Breteler MMB (1999). Diabetes mellitus and the risk of dementia. Neurology.

[B92] Park HJ, Kim J, Ryou C (2023). A three-dimensional spheroid co-culture system of neurons and
astrocytes derived from Alzheimer’s disease patients for drug efficacy
testing. Cell Prolif.

[B93] Pearson-Leary J, McNay EC (2016). Novel roles for the Insulin-regulated glucose transporter-4 in
hippocampally dependent memory. J Neurosci.

[B94] Petrovic D, Kouroussis E, Vignane T, Filipovic MR (2021). The role of protein persulfidation in brain aging and
neurodegeneration. Front Aging Neurosci.

[B95] Poetsch AR (2020). The genomics of oxidative DNA damage, repair, and resulting
mutagenesis. Comput Struct Biotechnol J.

[B96] Poprac P, Jomova K, Simunkova M, Kollar V, Rhodes CJ, Valko M (2017). Targeting free radicals in oxidative stress-related human
diseases. Trends Pharmacol Sci.

[B97] Rada P, Rojo AI, Chowdhry S, McMahon M, Hayes JD, Cuadrado A (2011). SCF/β-TrCP Promotes glycogen synthase kinase 3-dependent
degradation of the Nrf2 transcription factor in a Keap1-independent
manner. Mol Cell Biol.

[B98] Ramalingam M, Kim SJ (2014). The role of insulin against hydrogen peroxide-induced oxidative
damages in differentiated SH-SY5Y cells. J Recept Signal Transduct Res.

[B99] Ramsey CP, Glass CA, Montgomery MB, Lindl KA, Ritson GP, Chia LA, Hamilton RL, Chu CT, Jordan-Sciutto KL (2007). expression of Nrf2 in neurodegenerative diseases. J Neuropathol Exp Neurol.

[B100] Rojo AI, Pajares M, Rada P, Nuñez A, Nevado-Holgado AJ, Killik R, Van Leuven F, Ribe E, Lovestone S, Yamamoto M (2017). NRF2 deficiency replicates transcriptomic changes in Alzheimer’s
patients and worsens APP and TAU pathology. Redox Biol.

[B101] Rozumna NM, Hanzha VV, Lukyanetz EA (2023). Memantine protects the cultured rat hippocampal neurons treated
by NMDA and amyloid β1-42. Front Neurosci.

[B102] Sankar SB, Infante-Garcia C, Weinstock LD, Ramos-Rodriguez JJ, Hierro-Bujalance C, Fernandez-Ponce C, Wood LB, Garcia-Alloza M (2020). Amyloid beta and diabetic pathology cooperatively stimulate
cytokine expression in an Alzheimer’s mouse model. J Neuroinflammation.

[B103] Santiago JA, Bottero V, Potashkin JA (2019). Transcriptomic and network analysis highlight the association of
diabetes at different stages of Alzheimer’s disease. J Front Neurosci.

[B104] Scherer T, Sakamoto K, Buettner C (2021). Brain insulin signalling in metabolic homeostasis and
disease. Nat Rev Endocrinol.

[B105] Sharkus R, Thakkar R, Kolson DL, Constantinescu CS (2023). Dimethyl fumarate as potential treatment for Alzheimer’s disease:
Rationale and clinical trial design. Biomed.

[B106] Shi M, Chai Y, Zhang J, Chen X (2022). Endoplasmic reticulum stress-associated neuronal death and innate
immune response in neurological diseases. Front Immunol.

[B107] Shu J, Li N, Wei W, Zhang L (2022). Detection of molecular signatures and pathways shared by
Alzheimer’s disease and type 2 diabetes. Gene.

[B108] Shu XS, Zhu H, Huang X, Yang Y, Wang D, Zhang Y, Zhang W, Ying Y (2020). Loss of β-catenin via activated GSK3β causes diabetic retinal
neurodegeneration by instigating a vicious cycle of oxidative stress-driven
mitochondrial impairment. Aging (Albany NY).

[B109] Sims-Robinson C, Kim B, Rosko A, Feldman EL (2010). How does diabetes accelerate Alzheimer disease
pathology?. Nat Rev Neurol.

[B110] Singh B, Kumari S (2021). Assessment of correlation of oxidative stress and insulin
resistance with glucose-6-phosphate dehydrogenase activity in type ii
diabetes mellitus patients. J Pharm Bioallied Sci.

[B111] Steffan D, Pezzini C, Esposito M, Franco-Romero A (2025). Mitochondrial aging in the CNS: Unravelling implications for
neurological health and disease. Biomol.

[B112] Strong CE, Zhang J, Carrasco M, Kundu S, Boutin M, Vishwasrao HD, Liu J, Medina A, Chen YC, Wilson K (2023). Functional brain region-specific neural spheroids for modeling
neurological diseases and therapeutics screening. Commun Biol.

[B113] Su B, Wang X, Lee H gon, Tabaton M, Perry G, Smith MA, Zhu X (2010). Chronic oxidative stress causes increased tau phosphorylation in
M17 neuroblastoma cells. Neurosci Lett.

[B114] Sultana R, Mecocci P, Mangialasche F, Cecchetti R, Baglioni M, Butterfield DA (2011). Increased protein and lipid oxidative damage in mitochondria
isolated from lymphocytes from patients with Alzheimer’s disease: Insights
into the role of oxidative stress in Alzheimer’s disease and initial
investigations into a potential biomarker for this dementing
disorder. J Alzheimers Dis.

[B115] Sun LY (2006). Hippocampal IGF-1 expression, neurogenesis and slowed aging:
Clues to longevity from mutant mice. Age (Omaha).

[B116] Sun Y, Yang X, Xu L, Jia M, Zhang L, Li P, Yang P (2023). The Role of Nrf2 in Relieving Cerebral Ischemia-Reperfusion
Injury. Cur Neuropharmacol.

[B117] Sun Z, Kwon JS, Ren Y, Chen S, Walker CK, Lu X, Cates K, Karahan H, Sviben S, Fitzpatrick JAJ (2024). Modeling late-onset Alzheimer’s disease neuropathology via direct
neuronal reprogramming. Science.

[B118] Suzuki T, Takahashi J, Yamamoto M (2023). Molecular basis of the KEAP1-NRF2 signaling
pathway. Mol Cells.

[B119] Talbot K, Wang HY, Kazi H, Han LY, Bakshi KP, Stucky A, Fuino RL, Kawaguchi KR, Samoyedny AJ, Wilson RS (2012). Demonstrated brain insulin resistance in Alzheimer’s disease
patients is associated with IGF-1 resistance, IRS-1 dysregulation, and
cognitive decline. J Clin Invest.

[B120] Tamagno E, Guglielmotto M, Aragno M, Borghi R, Autelli R, Giliberto L, Muraca G, Danni O, Zhu X, Smith MA (2007). Oxidative stress activates a positive feedback between the γ- and
β-secretase cleavages of the β-amyloid precursor protein. J Neurochem.

[B121] Tang W, Li Y, He S, Jiang T, Wang N, Du M, Cheng B, Gao W, Li Y, Wang Q (2022). Caveolin-1 alleviates diabetes-associated cognitive dysfunction
through modulating neuronal ferroptosis-mediated mitochondrial
homeostasis. Antioxid Redox Signal.

[B122] Thubron EB, Rosa HS, Hodges A, Sivaprasad S, Francis PT, Pienaar IS, Malik AN (2019). Regional mitochondrial DNA and cell-type changes in post-mortem
brains of non-diabetic Alzheimer’s disease are not present in diabetic
Alzheimer’s disease. Sci Rep.

[B123] Thomassen JQ, Tolstrup JS, Benn M, Frikke-Schmidt R (2020). Type-2 diabetes and risk of dementia: observational and Mendelian
randomisation studies in 1 million individuals. Epidemiol Psychiatr Sci.

[B124] Tobin MK, Musaraca K, Disouky A, Shetti A, Bheri A, Honer WG, Kim N, Dawe RJ, Bennett DA, Arfanakis K (2019). Human hippocampal neurogenesis persists in aged adults and
Alzheimer’s disease patients. Cell Stem Cell.

[B125] Todorich B, Pasquini JM, Garcia CI, Paez PM, Connor JR (2009). Oligodendrocytes and myelination: The role of
iron. Glia.

[B126] Tönnies E, Trushina E (2017). Oxidative stress, synaptic dysfunction, and Alzheimer’s
disease. J Alzheimer’s Dis.

[B127] Traxler L, Lagerwall J, Eichhorner S, Stefanoni D, D’Alessandro A, Mertens J (2021). Metabolism navigates neural cell fate in development, aging and
neurodegeneration. Dis Model Mech.

[B128] Trujeque-Ramos S, Castillo-Rolón D, Galarraga E, Tapia D, Arenas-López G, Mihailescu S, Hernández-López S (2018). Insulin regulates GABAA receptor-mediated tonic currents in the
prefrontal cortex. Front Neurosci.

[B129] Trujillo CA, Gao R, Negraes PD, Gu J, Buchanan J, Preissl S, Wang A, Wu W, Haddad GG, Chaim IA (2019). Complex oscillatory waves emerging from cortical organoids model
early human brain network development. Cell Stem Cell.

[B130] Valdes P, Henry KW, Fitzgerald MQ, Muralidharan K, Caldwell AB, Ramachandran S, Goldstein LSB, Mobley WC, Galasko DR, Subramaniam S (2023). Limitations of the human iPSC-derived neuron model for
early-onset Alzheimer’s disease. Mol Brain.

[B131] Valko M, Leibfritz D, Moncol J, Cronin MTD, Mazur M, Telser J (2007). Free radicals and antioxidants in normal physiological functions
and human disease. Int J Biochem Cell Biol.

[B132] Van Der Heide LP, Kamal A, Artola A, Gispen WH, Ramakers GMJ (2005). Insulin modulates hippocampal activity-dependent synaptic
plasticity in a N-methyl-d-aspartate receptor and
phosphatidyl-inositol-3-kinase-dependent manner. J Neurochem.

[B133] Vanova T, Sedmik J, Raska J, Amruz Cerna K, Taus P, Pospisilova V, Nezvedova M, Fedorova V, Kadakova S, Klimova H (2023). Cerebral organoids derived from patients with Alzheimer’s disease
with PSEN1/2 mutations have defective tissue patterning and altered
development. Cell Rep.

[B134] Wang J, Jiang B, Lin X, Zhang J, Xiong L, Feng Y, San W, Xu B (2026). Free Radical Biology and Medicine. Genistein ameliorates
glucose-induced β -amyloid toxicity, oxidative stress, and aging in the C.
elegans model of Alzheimer’ s disease. Free Radic Biol Med.

[B135] Weissman L, Jo DG, Sørensen MM, de Souza-Pinto NC, Markesbery WR, Mattson MP, Bohr VA (2007). Defective DNA base excision repair in brain from individuals with
Alzheimer’s disease and amnestic mild cognitive impairment. Nucleic Acids Res.

[B136] Wodrich APK, Scott AW, Shukla AK, Harris BT, Giniger E (2022). The unfolded protein responses in health, aging, and
neurodegeneration: recent advances and future considerations. Front Mol Neurosci.

[B137] Wu XQ, Zhang DD, Wang YN, Tan YQ, Yu XY, Zhao YY (2021). AGE/RAGE in diabetic kidney disease and ageing
kidney. Free Radic Biol Med.

[B138] Xavier DJ, Takahashi P, Evangelista AF, Foss-Freitas MC, Foss MC, Donadi EA, Passos GA, Sakamoto-Hojo ET (2015). Assessment of DNA damage and mRNA/miRNA transcriptional
expression profiles in hyperglycemic versus non-hyperglycemic patients with
type 2 diabetes mellitus. Mutat Res Mol Mech Mutagen.

[B139] Xavier DJ, Takahashi P, Manoel-Caetano FS, Foss-Freitas MC, Foss MC, Donadi EA, Passos GA, Sakamoto-Hojo ET (2014). One-week intervention period led to improvements in glycemic
control and reduction in DNA damage levels in patients with type 2 diabetes
mellitus. Diabetes Res Clin Pract.

[B140] Yang YH, Wen R, Yang N, Zhang TN, Liu CF (2023). Roles of protein post-translational modifications in glucose and
lipid metabolism: mechanisms and perspectives. Mol Med.

[B141] Yao Y, Lu Q, Hu Z, Yu Y, Chen Q, Wang QK (2017). A non-canonical pathway regulates ER stress signaling and blocks
ER stress-induced apoptosis and heart failure. Nat Commun.

[B142] Zagare A, Kurlovics J, Almeida C, Ferrante D, Frangenberg D, Vitali A, Gomez-Giro G, Jäger C, Antony P, Halder R (2025). Insulin resistance compromises midbrain organoid neuronal
activity and metabolic efficiency predisposing to Parkinson’s disease
pathology. J Tissue Eng.

[B143] Zhang B, Pan C, Feng C, Yan C, Yu Y, Chen Z, Guo C, Wang X (2022). Role of mitochondrial reactive oxygen species in homeostasis
regulation. Redox Rep.

[B144] Zhang J, Yang H, Wu J, Zhang D, Wang Y, Zhai J (2022). Recent progresses in novel in vitro models of primary neurons: A
biomaterial perspective. Front Bioeng Biotechnol.

[B145] Zheng R, Zhang ZH, Chen C, Chen Y, Jia SZ, Liu Q, Ni JZ, Song GL (2017). Selenomethionine promoted hippocampal neurogenesis via the
PI3K-Akt-GSK3β-Wnt pathway in a mouse model of Alzheimer’s
disease. Biochem Biophys Res Commun.

[B146] Zhong Q, Xiao X, Qiu Y, Xu Z, Chen C, Chong B, Zhao X, Hai S, Li S, An Z (2023). Protein posttranslational modifications in health and diseases:
Functions, regulatory mechanisms, and therapeutic
implications. Med Comm.

[B147] Zhou Q, Ge X, Chen Z, Cao D, Chen Y, Shi J, Meng G (2025). Distinct types of protein modifications in diabetic endothelial
dysfunction. Cardiovasc Diabetol.

[B148] Zorov DB, Juhaszova M, Sollott SJ (2014). Mitochondrial reactive oxygen species (ROS) and ROS-induced ROS
release. Physiol Rev.

